# Supplementary material for: Valeriana rigida Ruiz & Pav. Root Extract: A New Source of Caffeoylquinic Acids with Antioxidant and Aldose Reductase Inhibitory Activities
Source: Foods. 2021 May 13;10(5):1079. doi: 10.3390/foods10051079 (PMC8152971; doi:10.3390/foods10051079)
Supplement: Supplementary file 1 [file foods-10-01079-s001.zip › foods-1205642-supplementary.pdf]

Supplementary Materials

Table 1. Antioxidant activity of the 70% MeOH root extract of *V. rigida* using DPPH, ABTS, and ORAC assays.

**Sample**	**TE (μmol Trolox equivalents per μg extract)**		
	**DPPH**	**ABTS**	**ORAC**
Extract	0.75 ± 0.05	0.82 ± 0.05	2.60 ± 0.08

Note: the antioxidant activity is expressed as Trolox equivalent antioxidant capacity (TE) (μmol Trolox equivalents/μg extract). Data was presented as mean ± SD ($n \geq 3$).

Table 2. Partition coefficients ($K_{upper/lower}$) of target compounds **1–7** in different solvent systems.

**Scheme 1.**	$K_{(upper/lower)}$ **value**						
	**1**	**2**	**3**	**4**	**5**	**6**	**7**
n-Hexane/EtOAc/MeOH/H_2O, 2:5:2:5	0.01	0.00	0.01	0.01	0.05	0.03	0.07
n-Hexane/EtOAc/MeOH/H_2O, 1:5:1:5	0.00	0.02	0.03	0.16	0.46	0.34	2.69
EtOAc/H_2O, 5:5	0.00	0.01	0.02	0.53	2.36	2.17	35.91
n-Hexane/EtOAc/MeOH/H_2O, 0.8:5:1.2:5	0.00	0.00	0.01	0.24	0.71	0.51	4.13
n-Hexane/EtOAc/MeOH/H_2O, 0.5:5:1.5:5	0.00	0.01	0.02	0.30	1.29	0.89	5.16
n-Hexane/EtOAc/MeOH/H_2O, 0.8:5:1.2:5	0.00	0.02	0.02	0.39	1.14	0.73	5.62
n-Hexane/EtOAc/MeOH/H_2O, 0.8:5:1.2:5+17.4 mM acetic acid	0.04	0.20	0.18	2.49	3.26	3.58	8.40
n-Hexane/EtOAc/MeOH/H_2O, 0.5:5:1.5:5+17.4 mM acetic acid	0.11	0.26	0.32	1.79	4.28	5.10	8.94
n-Hexane/EtOAc/MeOH/H_2O, 0.8:5:1.2:5+17.4 mM acetic acid	0.33	0.46	0.58	3.00	4.60	5.18	14.82

Table S3. ^{1}H-NMR chemical shifts (δ_H in ppm) and coupling constants (*J* in Hz) of the compounds **1–8** separated from the 70% MeOH extract of *V. rigida* root.

**No.**	**δ_H (*J* in Hz)**							
	**3-CQA (1)**	**4-CQA (3)**	**5-CQA (2)**	**3,4-diCQA (4)**	**3,5-diCQA (5)**	**4,5-diCQA (6)**	**3,4,5-triCQA (7)**	**Acacetin (8)**
2	1.86-2.02 (2H, m)	1.82-1.98 (2H, m)	1.78-2.00 (2H, m)	1.69-1.97 (2H, m)	1.74-2.12 (2H, m)	1.92-2.27 (2H, m)	2.00-2.29 (2H, m)	-
3	5.17 (1H, brs)	4.04 (1H, brs)	3.93 (1H, brs)	5.53 (1H, m)	5.15 (1H, d, 2.94)	4.30 (1H, d, 2.83)	5.42-5.44 (1H, m)	6.88 (1H, s)
4	3.54 (1H, brs)	4.68 (1H, dd, 7.44, 2.4)	3.56 (1H, d, 5.76)	5.06 (1H, brs)	3.71 (1H, dd, 9.72, 3.00)	5.09 (1H, dd, 10.15, 2.82)	5.21 (1H, dd, 7.74, 3.24)	-
5	3.87 (1H, brs)	3.98 (1H, d, 3.48)	5.08 (1H, brs)	3.77 (1H, brs)	5.34 (1H, ddd, 10.39, 10.38, 4.5)	5.69 (1H, ddd, 10.56, 10.53, 5.52)	5.42-5.44 (1H, m)	-
6	1.86-2.02 (2H, m)	1.82-1.98 (2H, m)	1.78-2.00 (2H, m)	1.69-1.97 (2H, m)	1.74-2.12 (2H, m)	1.92-2.27 (2H, m)	2.00-2.29 (2H, m)	6.21 (1H, d, 2.4)
8	-	-	-	-	-	-	-	6.51 (1H, d, 1.6)
2'	7.02 (1H, brs)	7.05 (1H, d, 1.38)	7.04 (1H, brs)	7.07 (1H, d, 1.74)	7.06 (1H, d, 1.02)	7.00 (1H, d, 1.20)	2' and 2'': 7.01 (2H, brs)	2' and 6': 8.04 (2H, d, 9.2)
2''	-	-	-	7.00 (1H, d, 1.80)	7.03 (1H, brs)	6.98 (1H, brs)		-
2'''	-	-	-	-	-	-	6.97 (1H, d, 0.96)	-
3'	-	-	-	-	-	-		7.11 (2H, d, 9.2)
5'	6.77 (1H, d, 7.33)	6.77 (1H, d, 8.10)	6.77 (1H, d, 8.10)	6.76 (1H, d, 8.10)	6.77 (1H, d, 7.92)	6.73 (1H, d, 8.16)	5' and 5'': 6.73 (2H, d, 8.04)	
5''	-	-	-	6.71 (1H, d, 8.22)	6.75 (1H, d, 7.80)	6.72 (1H, d, 8.16)		-
5'''	-	-	-	-	-	-	6.68 (1H, d, 8.10)	-
6'	6.96 (1H, d, 7.38)	7.00 (1H, dd, 8.16, 1.14)	6.98 (1H, d, 7.98)	7.03 (1H, dd, 8.10, 1.38)	6.98 (1H, dd, 8.04, 1.26)	6.89 (1H, dd, 8.22, 1.20)	6' and 6'': 6.95 (2H, brd, 8.16)	2' and 6': 8.04 (2H, d, 9.2)
6''	-	-	-	6.92 (1H, dd, 8.10, 1.68)	6.96 (1H, dd, 8.16, 1.50)	6.87 (1H, brd, 7.98)		-
6'''	-	-	-	-	-	-	6.91 (1H, brd, 8.28)	-
7'	7.46 (1H, d, 15.79)	7.50 (1H, d, 15.84)	7.42 (1H, d, 15.84)	7.48 (1H, d, 15.78)	7.49 (1H, d, 15.78)	7.57 (1H, d, 15.84)	7' and 7'': 7.46 (1H, d, 16.03)	-
7''	-	-	-	7.39 (1H, d, 15.84)	7.47 (1H, d, 15.78)	7.49 (1H, d, 15.84)	7.42 (2H, d, 15.37)	-
7'''	-	-	-	-	-	-		-
8'	6.19 (1H, d, 15.78)	6.27 (1H, d, 15.90)	6.16 (1H, d, 15.90)	6.32 (1H, d, 15.90)	6.24 (1H, d, 15.90)	6.23 (1H, d, 15.90)	6.20 (1H, d, 15.96)	-
8''	-	-	-	6.14 (1H, d, 15.84)	6.21 (1H, d, 15.90)	6.02 (1H, d, 15.84)	6.19 (1H, d, 15.96)	-
8'''	-	-	-	-	-	-	6.14 (1H, d, 15.85)	-
OCH3	-	-	-	-	-	-	-	3.86 (3H, s)

Note: the field strengths (MHz) and solvents used for ^{1}H-NMR checking are 600 MHz (DMSO-d_6) for 3-*O*-caffeoylquinic acid (3-CQA), 4-*O*-caffeoylquinic acid (4-CQA), 5-*O*-caffeoylquinic acid (5-CQA), 3,4-*O*-di-caffeoylquinic acid (3,4-diCQA), 3,5-*O*-di-caffeoylquinic acid (3,5-diCQA), and 3,4,5-*O*-tri-caffeoylquinic acid (3,4,5-triCQA); 600 MHz (MeOD-d_4) for 4,5-*O*-di-caffeoylquinic acid (4,5-diCQA); 400 MHz (DMSO-d_6) for acacetin. "-" means no signals appeared.
